# Supplementary material for: From sequence to enzyme mechanism using multi-label machine learning
Source: BMC Bioinformatics. 2014 May 19;15:150. doi: 10.1186/1471-2105-15-150 (PMC4229970; doi:10.1186/1471-2105-15-150)
Supplement: Additional file 2 — Java code of ml2db. Additional file ml2db_code.tar.gz contains the Java source code to run the multi-label machine learning experiments and save the results to database. The code’s Javadoc is included. [file 1471-2105-15-150-S2.zip › additional file 2/ml2db/utils/doc/index-files/index-4.html]

D-Index


JavaScript is disabled on your browser.


- Overview
- Package
- Class
- Use
- Tree
- Deprecated
- Index
- Help

- Prev Letter
- Next Letter

- Frames
- No Frames

- All Classes

A B C D E F G H I J K L M N O P Q R S T U V W X Y 


## D

Data - Class in uk.ac.standrews.utils.test
:   Class

Data() - Constructor for class uk.ac.standrews.utils.test.Data


DATA\_DB\_CONN\_PATH - Static variable in class uk.ac.standrews.utils.main.PathUtils


DATABASE\_NAME\_PROP - Static variable in class uk.ac.standrews.utils.main.database.DbConn


DATABASE\_TYPE\_PROP - Static variable in class uk.ac.standrews.utils.main.database.DbConn


DAY - Static variable in class uk.ac.standrews.utils.main.TimeUtils


DbConn - Class in uk.ac.standrews.utils.main.database
:   A DbConnectionProperties object contains the properties needed to connect to
    a database through JDBC.

DbConn(String) - Constructor for class uk.ac.standrews.utils.main.database.DbConn
:   Constructor: takes connection parameters from the specified properties
    file

DbConnectionSingleton - Class in uk.ac.standrews.utils.main.database
:   A singleton database connection

DbConnPropsTest - Class in uk.ac.standrews.utils.test.database
:   Class

DbConnPropsTest() - Constructor for class uk.ac.standrews.utils.test.database.DbConnPropsTest


DbCreator - Class in uk.ac.standrews.utils.main.database
:   Class

DbCreator(DbManager) - Constructor for class uk.ac.standrews.utils.main.database.DbCreator


DbCreatorTest - Class in uk.ac.standrews.utils.test.database
:   Class

DbCreatorTest() - Constructor for class uk.ac.standrews.utils.test.database.DbCreatorTest


DbManaged - Class in uk.ac.standrews.utils.main.database
:   Managed is a class which has a `DbManager` (an object that coordinates
    its database activities)

DbManaged(DbManager) - Constructor for class uk.ac.standrews.utils.main.database.DbManaged


DbManager - Class in uk.ac.standrews.utils.main.database
:   A database manager provides object to create a database, write and read from
    it.

DbManager(DbManager) - Constructor for class uk.ac.standrews.utils.main.database.DbManager
:   Constructor 2: creates the database manager from another one

DbManager(String) - Constructor for class uk.ac.standrews.utils.main.database.DbManager
:   Constructor 1: creates the database connection and the classes for
    reading/writing to database from database properties file

DbManagerSingleton - Class in uk.ac.standrews.utils.main.database
:   Class

DbManagerTest - Class in uk.ac.standrews.utils.test.database
:   Class

DbManagerTest() - Constructor for class uk.ac.standrews.utils.test.database.DbManagerTest


DbReader - Class in uk.ac.standrews.utils.main.database
:   Class to read from a database.

DbReader(DbManager) - Constructor for class uk.ac.standrews.utils.main.database.DbReader


DbReaderTest - Class in uk.ac.standrews.utils.test.database
:   Class

DbReaderTest() - Constructor for class uk.ac.standrews.utils.test.database.DbReaderTest


DbUtils - Class in uk.ac.standrews.utils.main.database
:   Database utilities (to create sql code etc.)

DbUtils() - Constructor for class uk.ac.standrews.utils.main.database.DbUtils


DbUtilsTest - Class in uk.ac.standrews.utils.test.database
:   Class

DbUtilsTest() - Constructor for class uk.ac.standrews.utils.test.database.DbUtilsTest


DbWriter - Class in uk.ac.standrews.utils.main.database
:   Provides methods to write to a database.

DbWriter(DbManager) - Constructor for class uk.ac.standrews.utils.main.database.DbWriter


DbWriterTest - Class in uk.ac.standrews.utils.test.database
:   Class

DbWriterTest() - Constructor for class uk.ac.standrews.utils.test.database.DbWriterTest


DEFAULT\_SEED - Static variable in class cern.jet.random.engine.MersenneTwister


DEFSEED - Static variable in class edu.cornell.lassp.houle.RngPack.Ranmar
:   Default seed.

DEFSEED1 - Static variable in class edu.cornell.lassp.houle.RngPack.Ranecu
:   default iseed1 = 12345

DEFSEED2 - Static variable in class edu.cornell.lassp.houle.RngPack.Ranecu
:   default iseed2 = 67890

deleteFile(String) - Static method in class uk.ac.standrews.utils.main.FileUtils
:   Delete a file

deleteKeyValue(T, U) - Method in class uk.ac.standrews.utils.main.maputils.OneToManyMap


deleteKeyValue(String, String) - Method in class uk.ac.standrews.utils.main.maputils.TableMap
:   Delete a key-value pair from the one to many map

depthFirstSearch(XmlNode, String, String, File) - Static method in class uk.ac.standrews.utils.main.webutils.simpledomparser.XmlSearcher
:   Searches this tree in a depth first mode and returns the first node whose
    tag, text or attribute value matches the given string.

depthFirstSearch(XmlNode, String, String, File, int, int) - Static method in class uk.ac.standrews.utils.main.webutils.simpledomparser.XmlSearcher
:   Searches this tree in a depth first mode and returns the first node whose
    tag, text or attribute value matches the given string.

depthFirstSearchOfParent(XmlNode, String, String) - Static method in class uk.ac.standrews.utils.main.webutils.simpledomparser.XmlSearcher


depthFirstSearchOfParent(XmlNode, String, String, File, int, int) - Static method in class uk.ac.standrews.utils.main.webutils.simpledomparser.XmlSearcher
:   TODO duplicated code! depthFirstSearch ~ depthFirstSearchOfParent
    Searches this tree in a depth first mode and returns the \*parent\* of the
    first node whose tag, text or attribute value matches the given string.

Diff - Class in uk.ac.standrews.utils.main.diff
:   Compares two collections, returning a list of the additions, changes, and
    deletions between them.

Diff(Collection, Collection) - Constructor for class uk.ac.standrews.utils.main.diff.Diff
:   Constructs the Diff object for the two collections, using the default
    comparison mechanism between the objects, such as `equals` and
    `compareTo`.

Diff(Collection, Collection, Comparator) - Constructor for class uk.ac.standrews.utils.main.diff.Diff
:   Constructs the Diff object for the two collections, using the given
    comparator.

Diff(Object[], Object[]) - Constructor for class uk.ac.standrews.utils.main.diff.Diff
:   Constructs the Diff object for the two arrays, using the default
    comparison mechanism between the objects, such as `equals` and
    `compareTo`.

Diff(Object[], Object[], Comparator) - Constructor for class uk.ac.standrews.utils.main.diff.Diff
:   Constructs the Diff object for the two arrays, using the given
    comparator.

diff() - Method in class uk.ac.standrews.utils.main.diff.Diff
:   Runs diff and returns the results.

Difference - Class in uk.ac.standrews.utils.main.diff
:   Represents a difference, as used in `Diff`.

Difference(int, int, int, int) - Constructor for class uk.ac.standrews.utils.main.diff.Difference
:   Creates the difference for the given start and end points for the
    deletion and addition.

DOUBLE - Static variable in class uk.ac.standrews.utils.main.database.SqlUtils
:   Basic data type: double (passes without error a parseDouble action)

DOUBLE\_QUOTE\_CHARACTER - Static variable in class uk.ac.standrews.utils.main.database.DbUtils


DOUBLE\_SQL\_DATATYPE - Static variable in class uk.ac.standrews.utils.main.database.DbUtils


doubleArrayToString(double[]) - Static method in class uk.ac.standrews.utils.main.collectionutils.ArrayUtils
:   Prints an array of objects

DoubleFunction - Interface in cern.colt.function
:   Interface that represents a function object: a function that takes a single
    argument and returns a single value.

doubleQuote(String) - Static method in class uk.ac.standrews.utils.main.stringutils.StringUtils
:   Enclose text in double quotes

doubleQuoteIfNeeded(String) - Static method in class uk.ac.standrews.utils.main.stringutils.StringUtils
:   Enclose text in double quotes if it contains non-alphanumerical
    characters such as .

doubleTo2DecimalsString(Double) - Static method in class uk.ac.standrews.utils.main.NumberUtils
:   Get a percentage representation of a double

dropCreateTable() - Method in class uk.ac.standrews.utils.main.database.TableCreator
:   Drops and creates table corresponding to this Table object.

dropTable(String) - Method in class uk.ac.standrews.utils.main.database.DbCreator
:   Drops the database table.

dropTable() - Method in class uk.ac.standrews.utils.main.database.TableCreator
:   Drops the database table for this Table object.

A B C D E F G H I J K L M N O P Q R S T U V W X Y

- Overview
- Package
- Class
- Use
- Tree
- Deprecated
- Index
- Help

- Prev Letter
- Next Letter

- Frames
- No Frames

- All Classes
